# Supplementary figures and images for: mRNA stability fine-tunes gene expression in the developing cortex to control neurogenesis
Source: PLoS Biol. 2025 Feb 6;23(2):e3003031. doi: 10.1371/journal.pbio.3003031 (PMC11838918; doi:10.1371/journal.pbio.3003031)

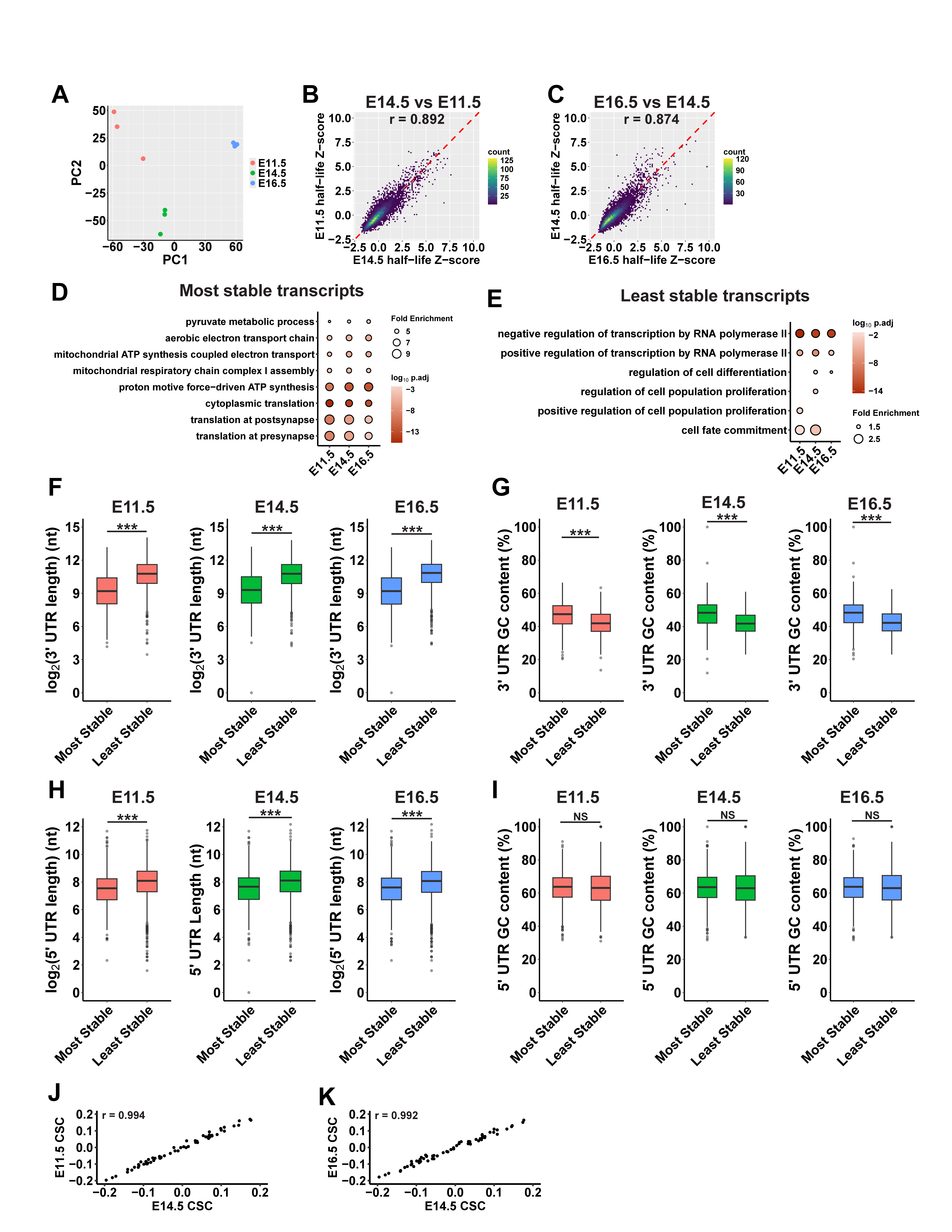

Supplement: S1 Fig — (A) Principal component analysis of biological replicates from SLAM-seq half-life data. (B) Correlation between z-score normalized half-lives at E11.5 and E14.5. Red dashed line indicates y = x line; r represents the Pearson correlation coefficient. (C) As in B, for E14.5 and E16.5. (D and E) GO analysis of top 10% most stable and least stable transcripts. Circle size represents fold enrichment and color represents adjusted p-value. (F) Comparison of 3′ UTR lengths of the top 10% most and least stable transcripts at each stage. (G) As in E, comparing percentage GC content. (H, I) As in F, G, for 5′ UTRs. (J) Comparison of CSC values between E11.5 and E14.5; r represents the Pearson correlation coefficient. (K) As in J, for E14.5 and E16.5. ***p < 0.001. Wilcoxon rank-sum test (F, H), Welch’s two-sample t test (G, I). Underlying data for this figure can be found in S9 Data. (TIF) [file pbio.3003031.s001.tif]

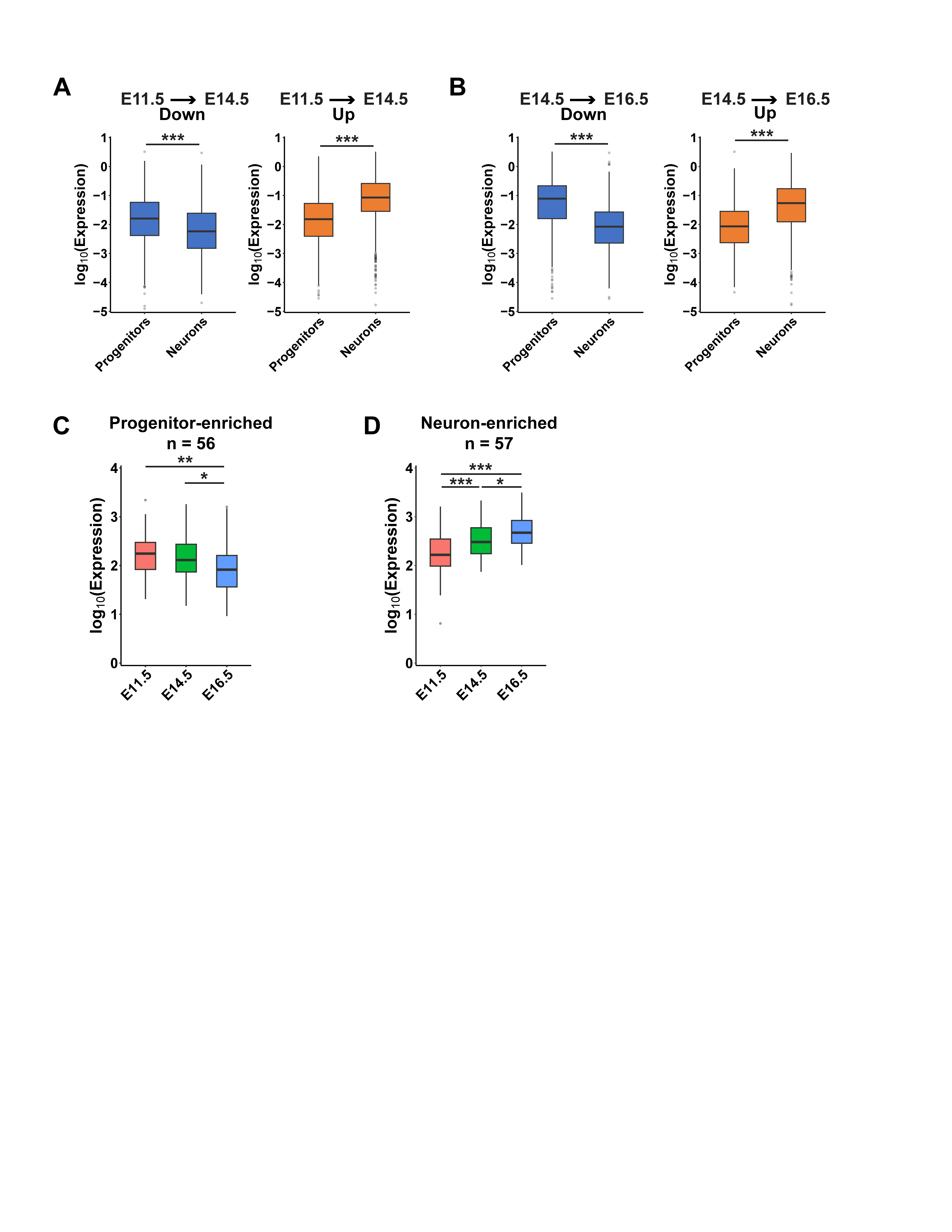

Supplement: S2 Fig — (A) Expression of differentially expressed genes at E14.5 compared to E11.5. Expression data from publicly available scRNA-seq data sets [6]. (B) As in A, for differentially expressed genes at E16.5 compared to E14.5. (C) Expression of progenitor-enriched genes across development using SLAM-seq expression data from this study. (D) As in C, for neuron-enriched genes. *p < 0.05, ***p < 0.001. Wilcoxon rank-sum test (A, B), one-way ANOVA with Tukey’s HSD post hoc test (C, D). Underlying data for this figure can be found in S10 Data. (TIF) [file pbio.3003031.s002.tif]

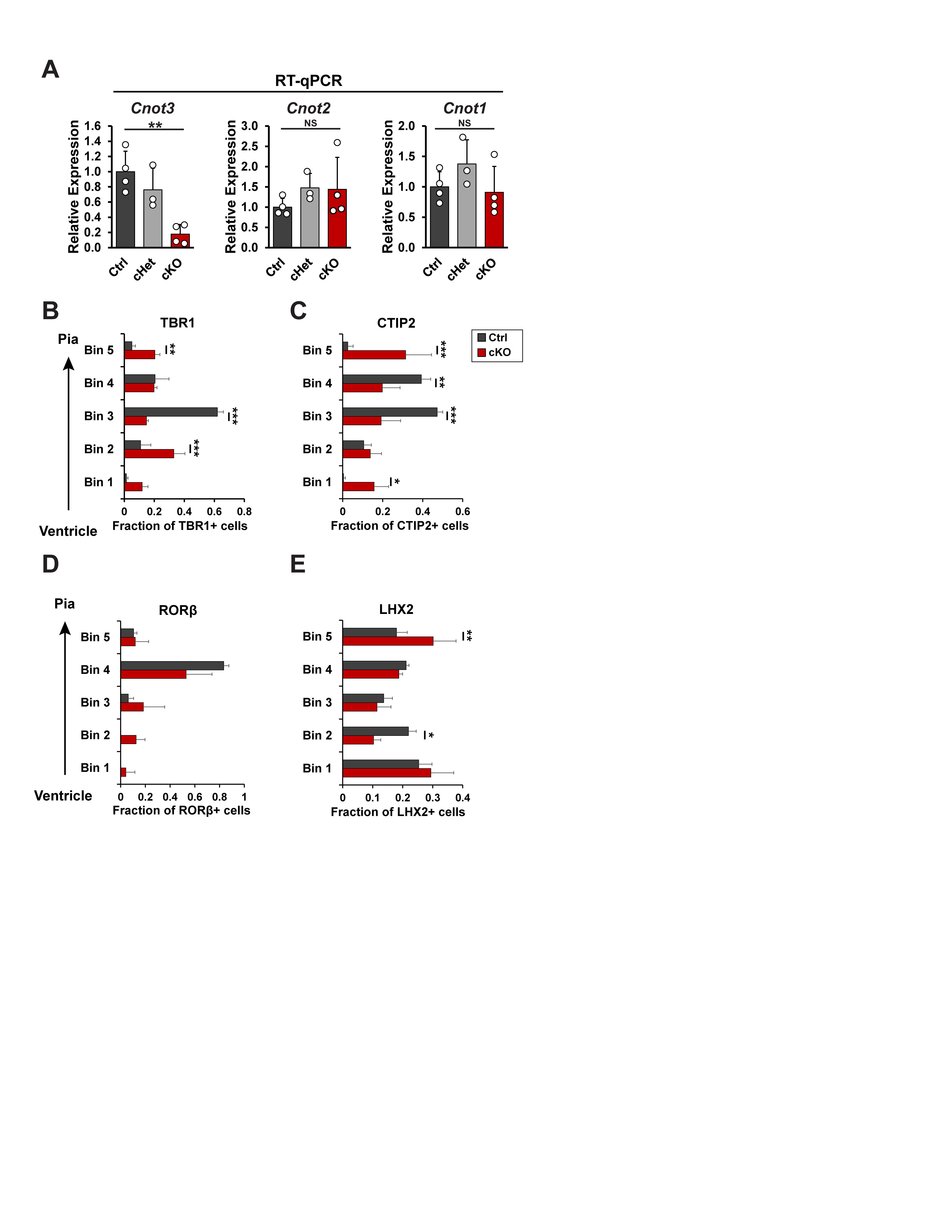

Supplement: S3 Fig — (A) Expression of Cnot1, Cnot2, and Cnot3 in E12.5 cortical lysates measured by RT-qPCR. (B–D) Distribution of indicated marker(s) at E18.5 across 5 equally sized cortical bins spanning the ventricular surface (Bin 1) to the pia (Bin 5). *p < 0.05, **p < 0.01, ***p < 0.001. One-way ANOVA with Tukey’s HSD post hoc test (A), two-way ANOVA with Tukey’s HSD post hoc test (B–D). Error bars represent standard deviation. Underlying data for this figure can be found in S11 Data. (TIF) [file pbio.3003031.s003.tif]

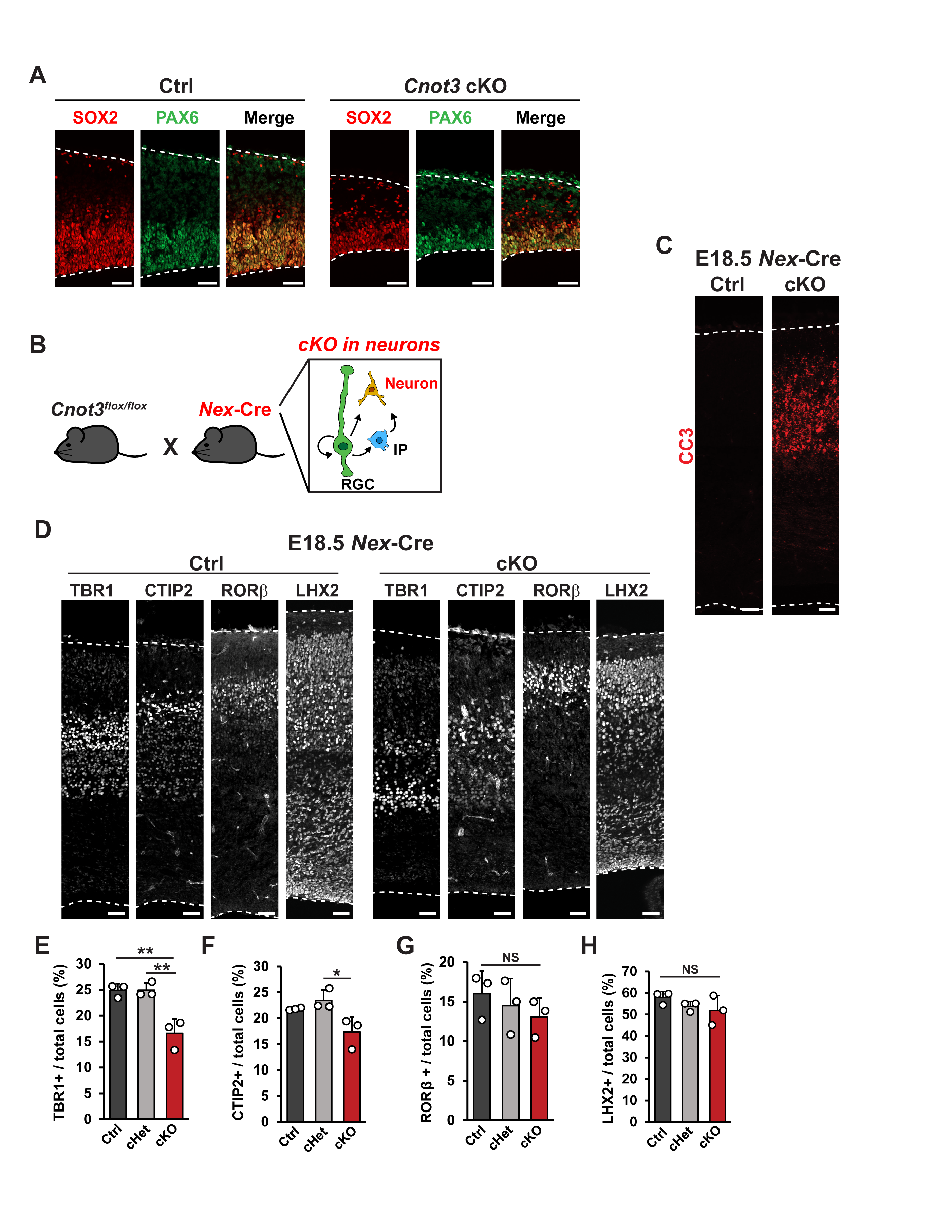

Supplement: S4 Fig — (A) Immunofluorescence against SOX2 and PAX6 in E14.5 control and Emx1-Cre cKO cortices. (B) Schematic showing strategy for Cnot3 cKO in neurons using Nex-Cre. (C) Immunofluorescence against CC3 in E18.5 control and Nex-Cre cKO cortices. (D) Immunofluorescence against indicated marker(s) in E18.5 control and Nex-Cre cKO cortices. (E–H) Quantification of density for the indicated markers (n = 3 embryos per genotype). *p < 0.05,**p < 0.01. One-way ANOVA with Tukey’s HSD post hoc test (F–H). Error bars represent standard deviation. Scale bars: 50 μm for all images. Underlying data for this figure can be found in S13 Data. (TIF) [file pbio.3003031.s004.tif]

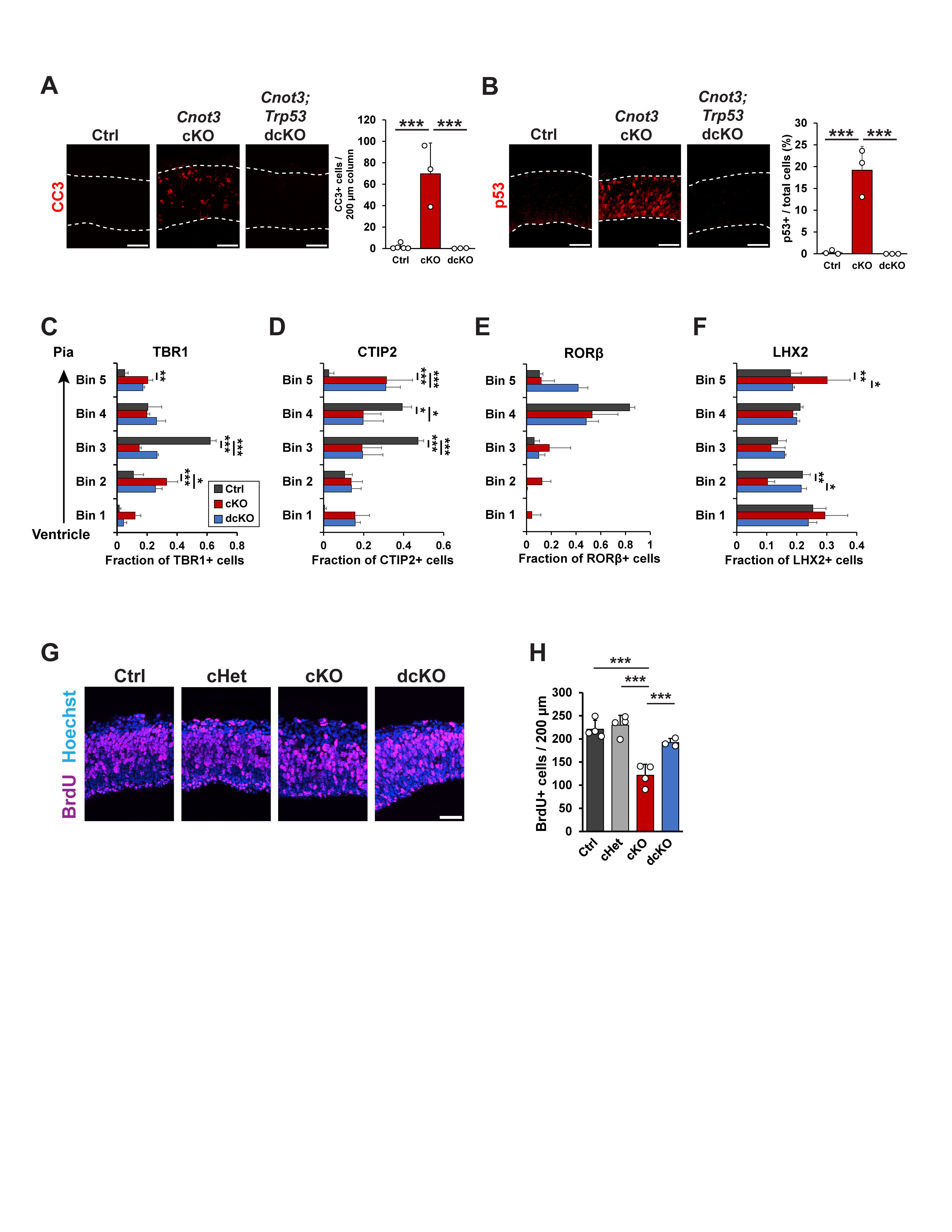

Supplement: S5 Fig — (A) Representative images of immunofluorescence against CC3 in E12.5 cortices showing rescue of apoptosis in dcKO mice. (B) Representative images of immunofluorescence against p53 in E12.5 cortices showing rescue of p53 accumulation in dcKO mice. (C–F) Distribution of indicated marker(s) at E18.5 across 5 equally sized cortical bins spanning the ventricular surface (Bin 1) to the pia (Bin 5). (G) Representative images showing E12.5 cortices of the indicated genotype pulse labeled by IP injection of BrdU 2 h prior to dissection. (H) Quantificaiton of BrdU+ cells (n = 3–4 embryos per genotype). *p < 0.05, **p < 0.01, ***p < 0.001. Three-way ANOVA with Tukey’s HSD post hoc test (C–F). One-way ANOVA with Tukey’s HSD post hoc test (H). Error bars represent standard deviation. Scale bars: 50 μm (A, B, G). Underlying data for this figure can be found in S13 Data. (TIF) [file pbio.3003031.s005.tif]

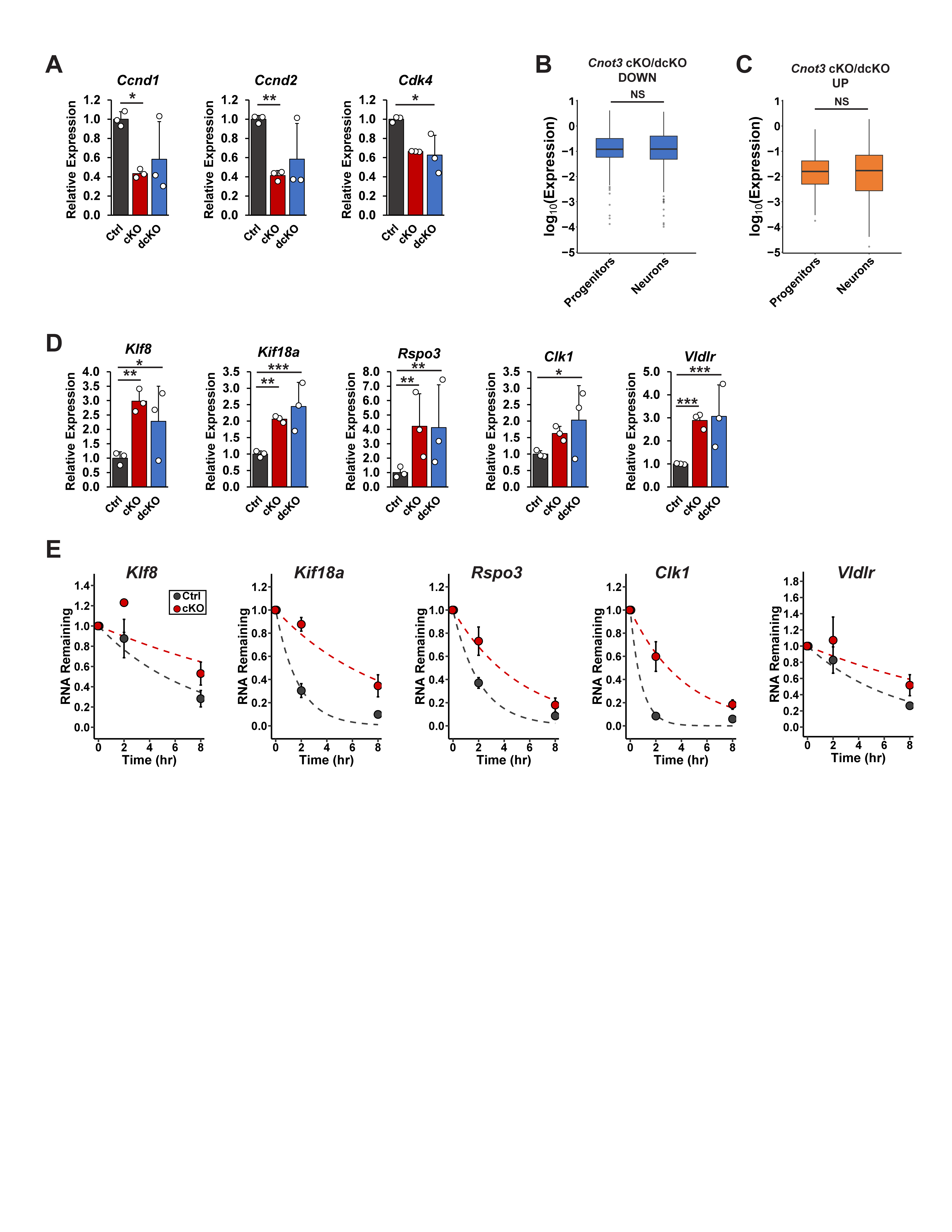

Supplement: S6 Fig — (A) Expression of the indicated cell cycle genes at E12.5 measured by RNA-seq. (B) Expression levels of transcripts that are down-regulated in both Cnot3 cKO and dcKO in either progenitors or neurons. Expression data taken from ref. [6]. (C) As in (B), for transcripts that are up-regulated in both cKO and dcKO cortices. (D) Transcript levels at E12.5 measured by RNA-seq. (E) Decay curves measured by RT-qPCR following transcriptional shut-off in E12.5 primary cultures. Dashed lines indicate fit to exponential decay equation. *p < 0.05, **p < 0.01, ***p < 0.001. Values shown are adjusted p-values from DESeq2 (A, D). Error bars represent standard deviation. Underlying data for this figure can be found in S14 Data. (TIF) [file pbio.3003031.s006.tif]

## Western blots used for Figure 4C

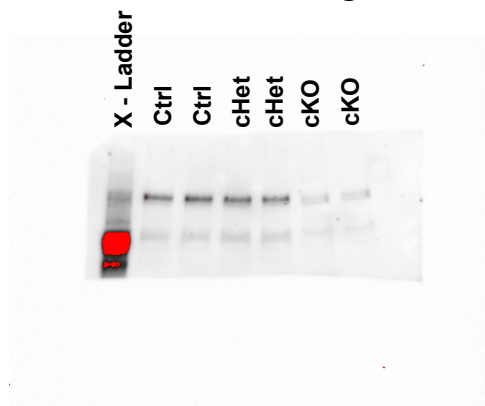

**CNOT3**

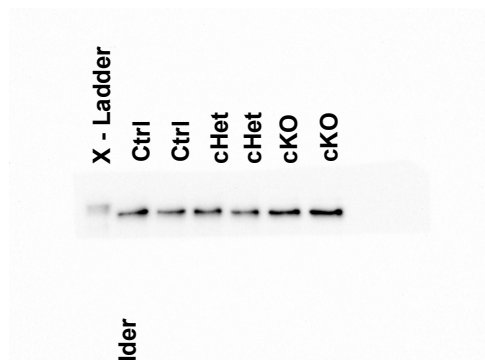

**CNOT2**

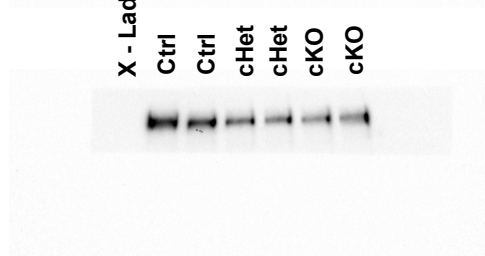

**CNOT1**

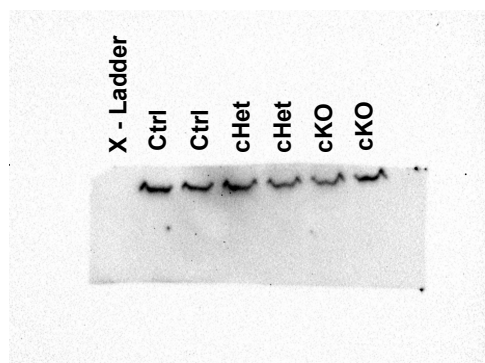

**ACTB**

Supplement: S1 Raw Images — (PDF) [file pbio.3003031.s030.pdf]
