# Supplementary material for: mRNA stability fine-tunes gene expression in the developing cortex to control neurogenesis
Source: PLoS Biol. 2025 Feb 6;23(2):e3003031. doi: 10.1371/journal.pbio.3003031 (PMC11838918; doi:10.1371/journal.pbio.3003031)
Supplement: Attachment — Submitted filename: Response to Reviewers.pdf [file pbio.3003031.s031.pdf]

**Reviewer #1**: The Silver lab has a long and impressive history in studying cortical development with a special emphasis on neural progenitors. In the current paper, they take on to systematically identify the landscape of RNA stability in the developing task using SLAM-seq, a by now well established approach in RNA biology. This allowed them to identify the most and least stable transcripts, respectively, for each time point of mouse development and subsequently analyze their 3'-UTR in detail. Rewardingly, but not surprisingly, this careful and well-planned analysis yielded distinct clusters of transcripts with different dynamics at different time points, which is the promising basis for this important study. The importance and the quality of this data set is clearly shown in the provided Volcano plots in Fig. 3, identifying known markers for neuronal development, e.g. Neurod2, Dcx, Hes5, Sox2. Also, progenitor-enriched transcripts are often rather unstable compared to their neuron-enriched counterparts arguing for cell-specific layers of RNA stability. Next, the authors turned to CNOT3, a central component of the CCR4-NOT complex important for deadenylation in cells, with a known role in neurodevelopmental diseases. Taking advantage of their impressive mouse genetics expertise, they decided to target radial glia and their progeny, e.g. IPCs and neurons, by using the existing Emx1-Dre driver line and characterized the resulting transgene in great detail. Interestingly, their cKO mice exhibited profound microcephaly with significant reduction in cortical thickness, with a particular impact on glutamatergic neurons. In particular, Cnot3 cKO mice have early defects during mid-neurogenesis arguing for an early role of CNOT3 affecting both RGCs and IPCs. Consequently, apoptosis was found to take place in these mice as judged by cleaved caspase 3 staining and by accumulation of p53, an important signaling molecule for apoptosis. Rescue of p53-dependent apoptosis led to a clear rescue of cortical size. Consistent with this is their finding that the loss of upper layer neurons (but not their lower layer counterpart) is dependent on p53. Using a clever in vivo semicumulative labeling assay, they found that Cnot3 loss yielded an overall slower cell cycle consistent with their previous findings. Most impressively, when performing a live imaging assay to monitor cell fates showed that progenitors undergo fewer neurogenic divisions in CKO cells arguing for impaired neuron generation. Obviously, the authors wanted to know on which targets CNOT3 might act and therefore performed bulk RNA-seq from their various mouse lines yielding large lists of potential RNA targets. Here, there is evidence that CNOT3 is likely to control expression of poorly expressed, non-optimal mRNAs (based on codon usage) in the cortex.

Overall, the work is of usual high quality, very important for a broad community with important biological implications. The data in the presented figures are impressive, important and the experiments well described. Please find some comments for the authors to consider before publication.

We thank the reviewer for their positive feedback and are pleased that they find the work “impressive” and “important”.

I do not know about the history of this important study. It may have seen the eyes of reviewers before and possibly their suggestions have impacted on the current manuscript. I am not sure that the inclusion of Figure 8 really helps the consistency of the study. Needless to say that the identification of important targets of CNOT3 would be a big advance. However, I am not convinced that the current list of data is consistent, logical and of functional importance yet. If this set of data stays in, the authors might want to focus on telling us, which of the identified transcripts might be functionally relevant here. At least, a more coherent view on what has been

done would help the reader.

We thank the reviewer for this important suggestion. We have made several changes to the text in the Results section pertaining to the RNA-seq data in Figure 8 to further emphasize the functional significance of our findings, and to improve the logical flow of the data. We have also added new analyses investigating cell type-specific expression levels of *Cnot3*-sensitive transcripts, by comparing our data with previously published datasets (Supplemental Fig. S6B, C). These analyses showed that differentially expressed transcripts were not biased for expression in either progenitors or neurons. This illustrates how our RNAseq data can be mined to understand new mechanisms of *Cnot3* function. We also chose to remove the GO analysis of these transcripts as the results did not provide a meaningfully important addition to the functional role of *Cnot3* in the cortex. In sum, these RNA-seq datasets provide molecular support for cell cycle phenotypes reported above. Furthermore, these data suggest that CNOT3 plays a role in helping to suppress a subset of transcripts that are typically expressed at low levels across multiple cell types.

Further comments for the authors

**1.** Enlarge panel A in Fig. 1 to do justice to this important scheme

We agree and have enlarged Fig. 1A as suggested.

2. Possibly combine Figs 2. And 3, e.g. the key panels.

We understand the suggestion, however we feel these two figures make distinct points and prefer to keep them separate, since we are not limited by figure count.

3. Provide your rationale for why to focus on CNOT3! Indicate this gene in your previous plots and tell us the evidence?

We thank the reviewer for this suggestion. The following rationale for focusing on CNOT3 has been added to the results section. First, our SLAM-seq data indicated that codon optimality is highly correlated with mRNA stability in the embryonic cortex (Fig. 1G). Mechanistically, codon content and its effects on translation are monitored by the CCR4-NOT complex to drive degradation of non-optimal mRNAs. This led us to investigate the *in vivo* consequences of mis-regulating the CCR4-NOT complex during cortical development. To address this, we focused on CNOT3, an essential component of the complex. Second, mutations in *CNOT3* are associated with neurodevelopmental disorders in humans, however its role in cortical development was unknown prior to this work.

4. Improve content of scheme Fig. 4A

We have modified Fig. 4A to emphasize the role of the CCR4-NOT complex in deadenylation and mRNA degradation.

5. Show video (or a sequence of stills) from live cell imaging data in Fig. 8

In the revised submission we have added videos from the live imaging data depicting examples of proliferative and neurogenic divisions (Supplemental Movies S1, S2)

**Reviewer #2** (Yi-Shuian Huang, signs review): Serdar et al. reported transcriptome-wide alterations in RNA stability during cortical development and highlighted how molecular defects in RNA degradation contribute to abnormal cortical development in CNOT3-cKO mice. Through metabolic labeling of cells isolated from cortices at E11.5, E14.5, and E16.5, their SLAM-seq analysis uncovered several key omics principles, including that increased RNA stability is correlated with shorter 5'-UTR and 3'-UTR lengths, reduced m6A modifications, and higher usage of optimal codons. Although dysregulation of RNA turnover is associated with neurodevelopmental disorders, its specific role in cortical development remains largely unclear. Given that mutations in CNOT1 and CNOT3, both components of the CCR4-NOT complex, are associated with autism and developmental disabilities, the authors opted for conditional ablation of CNOT3 in Emx1-lineage cells to investigate the resulting defects in cortical development. Notably, RNAs that are upregulated during development tend to exhibit greater stability, while those that are downregulated are generally less stable. Together, their findings demonstrate that precise regulation of RNA turnover is essential for proper brain development. This is a well-done and excellent study. I only have a few questions I would like the authors to clarify.

We thank the reviewer for their careful analysis of the manuscript and are pleased that they find the work “well done and excellent”.

Specific Comments:

1. What is the cortical size in CNOT3-heterozygous mice? To better reflect the patient situation, one would expect cortical development to be impaired in these mice. Can the authors provide at least a basic analysis of cortical thickness and layer thickness in CNOT3-heterozygous mice? If this analysis is not feasible within the revision timeline (assuming they do not keep global heterozygous knockout mice), further discussion connecting their findings to clinical patients should be elaborated.

We thank the reviewer for this important suggestion and agree that a germline mouse may further model how nonsense heterozygous mutations in *CNOT3* contribute to human disease. Unfortunately, we do not maintain global *Cnot3* heterozygous mice and therefore generating and analyzing them would extend beyond the timeline of this resubmission. As suggested by the reviewer, we have extensively revised the final paragraph of the Discussion section to elaborate on the relationship between our data and known clinical phenotypes in patients. Importantly, emerging work indicates that many autism risk and intellectual disability genes frequently converge on dysregulation of neurogenesis including development of deep layer neurons. Given our phenotypes, this suggests that aberrant cortical development likely is an important contributor to neurodevelopmental pathologies associated with *CNOT3* mutation. Further study of this cKO mouse model may thus provide important insights into the etiology of *CNOT3* pathologies. We also agree that an important next step for future studies will be to investigate how germline nonsense and missense mutations of *Cnot3* impact the developing cortex.

2. In Fig 5E, there are only a few CC3-positive cells, but they should be detected in the

control cortex. Could the authors provide quantified data for this, since they have images already?

As suggested, we have quantified CC3 positive cells for the conditions shown in Fig. 5E and have included this data as a new graph in the revised manuscript (Fig. 5F). In addition, we have also included new quantification of CC3 and p53 positive cells in cKO vs dcKO cortices showing the rescue of p53-dependent apoptosis in the dcKO condition (Supp. Fig. S5A, B).

3. Interestingly, the conditional ablation of p53 rescues neurons in the superficial layers but not in layers 5-6. The authors suggest that the loss of upper layer neurons is p53-dependent, whereas the loss of deep layer neurons is largely p53-independent. Could it be possible that proliferation plays a more significant role than apoptosis in the deeper layer neurons? If so, what is the number of BrdU-labeled cells in E12.5 dcKO cortex compared to those in Ctrl and CNOT3-cKO cortices shown in Fig. 7A?

To test whether proliferation is altered in dcKO cortices, we quantified proliferating cells with BrdU at E12.5, as suggested by the reviewer. The results of this have been added to the revised manuscript (Supplemental Figure S5G, H). In dcKO brains we observed rescue of proliferative cell number suggesting p53 contributes to E12.5 RGC number. However, in contrast, loss of deep layer neurons at E18.5 is not rescued by p53. This suggests there must be apoptotic-independent mechanisms at play.
Importantly, our semi-cumulative labeling and live imaging data in cKO cells (Fig 7) demonstrate that E12.5 progenitors take longer to divide and tend to produce more progenitors at the expense of neurons. At E12.5 the primary cortical neurons being produced are deep layer. This suggests that changes in cell cycle and cell fate are important contributors to reduction of deep layer neurons in dcKO cortices. Thus we conclude both apoptosis and proliferation contribute to *Cnot3* phenotypes. Future studies will be needed to tease out these important functions in detail. We have also revised our model to more carefully define *Cnot3* phenotypes and functions (Fig. 9).

4. Please deposit the NGS data and provide the GSE number in the revised manuscript.

Sequencing data has been deposited on GEO with the accession numbers GSE281690 (Cnot3 RNA-seq) and GSE281693 (SLAM-seq). The Data Availability section of the manuscript has been updated to reflect this.

The data will remain private prior to publication, however reviewers may access the data using the following tokens:

- GSE281690: ofyfgqmytfytvsx
- GSE281693: opavoiokhzudvyv
